# AMPING UP HEALTHY AGING: ENHANCING THE LIVES OF OLDER ADULTS AMONG DIVERSE POPULATIONS

**DOI:** 10.1093/geroni/igae098.1699

**Published:** 2024-12-31

**Authors:** Ginger Williams, Sue Schneider, Eric Ishiwata, Sara Delgado, Peggy Stoltenberg, Christine Fruhauf, Allyson Brothers

**Affiliations:** Colorado State University, Deer Trail, Colorado, United States; Colorado State University, Fort Collins, Colorado, United States; Colorado State University, Fort Collins, Colorado, United States; Colorado State University, Fort Collins, Colorado, United States; Colorado State University, Fort Collins, Colorado, United States; Colorado State University, Fort Collins, Colorado, United States; Colorado State University, Fort Collins, Colorado, United States

## Abstract

Over the last decade, Colorado State University Extension (CSUE) has focused on meeting the needs of historically marginalized communities with the highest rates of health disparities and social isolation. These disparities are largely due to language barriers and gaps in cultural competencies among healthcare providers. To bring healthy aging education and support to culturally diverse and underserved communities, CSUE led the adaptation of National Council on Aging’s (NCOA) Aging Mastery Program (AMP), a ten-session evidence-based curriculum (available in both English and Spanish) that supports older adults in aging well. The adaptation of AMP was implemented with Native American, Spanish-dominant immigrant, and East African refugee populations in Colorado. To adapt the program, we utilized state data to identify priority communities. Next, we conducted listening sessions to understand each community’s unique perspectives with a significant focus on developing trusting relationships. We then identified and supported members of each community as champions to teach the curriculum with CSUE’s coaching and technical assistance. Finally, we empowered these champions and their class participants to identify other gaps in knowledge and areas of interest, creating opportunities to develop ‘booster’ sessions. We prioritized paying our community champions to teach and lead in their communities. As a result, we’ve co-written grants and papers and created new programs alongside these champions. We are currently exploring expanding AMP in eight new communities across Colorado. Our program adaptation process has helped to break down barriers to implementing culturally relevant programs with underserved communities while bridging health equity gaps across the state.

